# Data mining of the GAW14 simulated data using rough set theory and tree-based methods

**DOI:** 10.1186/1471-2156-6-S1-S133

**Published:** 2005-12-30

**Authors:** Liang-Ying Wei, Cheng-Lung Huang, Chien-Hsiun Chen

**Affiliations:** 1 Institute of Biomedical Sciences, Academia Sinica, Huafan University, Taipei, Taiwan; 2 Department of Information Management, Huafan University, Taipei, Taiwan; 3 Department of Information Management, National Kaohsiung First Universityof Science and Technology, Kaohsiung, Taiwan; 4 College of Chinese Medicine, China Medical University, Taichung, Taiwan

## Abstract

Rough set theory and decision trees are data mining methods used for dealing with vagueness and uncertainty. They have been utilized to unearth hidden patterns in complicated datasets collected for industrial processes. The Genetic Analysis Workshop 14 simulated data were generated using a system that implemented multiple correlations among four consequential layers of genetic data (disease-related loci, endophenotypes, phenotypes, and one disease trait). When information of one layer was blocked and uncertainty was created in the correlations among these layers, the correlation between the first and last layers (susceptibility genes and the disease trait in this case), was not easily directly detected. In this study, we proposed a two-stage process that applied rough set theory and decision trees to identify genes susceptible to the disease trait. During the first stage, based on phenotypes of subjects and their parents, decision trees were built to predict trait values. Phenotypes retained in the decision trees were then advanced to the second stage, where rough set theory was applied to discover the minimal subsets of genes associated with the disease trait. For comparison, decision trees were also constructed to map susceptible genes during the second stage. Our results showed that the decision trees of the first stage had accuracy rates of about 99% in predicting the disease trait. The decision trees and rough set theory failed to identify the true disease-related loci.

## Background

Data mining approaches have been applied to different areas to derive useful and comprehensive knowledge. Methods focusing on functionalities of data mining, such as classification, prediction, association, and clustering, have been developed [1]. Variants of decision trees, such as ID3 [2] and C4.5 [3], have become standard tools for classification [4,5]. Recently, tree-based methods have been applied to genome-wide association studies for disease gene mapping [6]. Rough set theory [7] has also been utilized to solve decision problem in business and industrial areas [8-10]. In this study, we proposed two-stage methods that utilize decision trees C4.5 and rough set theory to analyze the Genetic Analysis Workshop 14 (GAW14) simulated data. Our goal was to search genes susceptible to Kofendrerd Personality Disorder (KPD), a behavioral disorder with multiple possible phenotype definitions.

## Methods

### Materials

The GAW14 simulated data was generated to represent diseased families sampled from four geographically diverse sites, Aipotu, Karangar, Danacaa, and New York City, with varied criteria for diagnosis of KPD. Subjects from these four sites had different living environments and ethnic backgrounds. One hundred replicates were generated. In each replicate, 100 nuclear families were collected from each of the first three sites and 50 extended families from the fourth site. In addition to the KPD affected status, 12 KPD-related phenotypes, labeling as *a*, *b*, *c*, ...,*l*, were given for each subject. A total of 917 SNP markers, spaced 3 cM apart, were provided on 10 chromosomes. In addition, a genome screen of 416 microsatellite markers, spaced 7 cM apart, was also given. In this study, only the SNP datasets of the first 10 replicates were analyzed. Simulated data answers were revealed after the analysis was done.

### Decision trees

A decision tree is often constructed based on some attributes to divide a group of subjects into more homogenous subgroups with respect to the target outcome variable. Briefly, a decision tree is built using a recursive partitioning process and a pruning process. Initially, a root node is built to represent the entire group. Then two leaf nodes are constructed, each representing a subgroup with a specific character of a selected attribute. At each level of tree construction, entropy was employed to calculate the information gain of each attribute. The attribute with the maximal information gain was chosen as a node at that level. The process continued until we got to the end of the branch. Then, each branch was defined as the leaf of the selected attribute. A route stemmed from the root to the leaf is defined as a rule. The attribute closer to the tree root is the most important decision factor for the rule. The pruning process for a decision tree was to replace a whole sub-tree with a leaf node. The replacement took place if a decision rule was established such that the expected error rate in the sub-tree was greater than in the single leaf. With this approach, the final decision tree was built. In this study, the C4.5 Release 8 software  was used to build the decision tree. The choice of pruning confidence affects how the error rates were estimated and hence the severity of pruning; values smaller than the default (25%) cause more of the initial tree to be pruned, while larger values resulted in less pruning. In this study, the pruning confidence level was set at 25%. The GAW14 simulated data was transformed into an appropriate format for the software. The KPD affected status and the 12 phenotypes were coded as 0 and 1 for unaffected and affected; SNP genotypes 11, 12, and 22 were coded as 1, 2, and 3, respectively.

### Rough set theory

Rough set theory (RST), introduced by Pawlak [7], has been widely investigated in areas such as machine learning, knowledge acquisition, decision analysis, knowledge discovery, and pattern recognition [8,10]. A simple example is used to illustrate the RST procedure. An eight-subject dataset is coded as in the raw data part of Table 1. Four conditional attributes (the genotypes of four single-nucleotide polymorphisms (SNPs)) and one decision variable (affected status) are included and denoted as A = {a1, a2, a3, a4, D}. It is easy to see that there are two classes in Table 1: Class 1 = {X_1_, X_4_, X_6_, X_7_} for D = 1 and Class 2 = {X_2_, X_3_, X_5_, X_8_} for D = 2. The set of attributes that discerns the elementary set {X_1_, X_2_} contains attribute a2 and a4, which will be put into the discernibility matrix (Table 2). Because we are not interested in the set of attributes that discern these four objects in Class 1, the corresponding cells in the discernibility matrix will be presented using " -". The discernibility matrix is then used to find the minimal subsets of the attributes by calculating a discernibility function as following:

*f*_*A *_(*D*) = (a2, a4)(a1,a2,a4)(a1, a4)(a1,a2,a3)(a1,a2) (a1,a2,a4)(a2,a3,a4)(a1,a2,a4)

(a1,a2,a4)(a1,a2,a3)(a2)(a1,a2,a3,a4)(a2,a4)(a1,a2,a3,a4)(a1,a2,a3)(a1,a2,a4)

= (a1, a4) a2 = a1a2+a2a4

The so-called discernibility function *f(A) *is a Boolean function, constructed as follows: a1a2 represented as a_1 _∧ a_2_, i.e., a1 and a2, and (a1, a2) represented as (a1 + a2) or it can be represented as (a1 or a2). The functions a1a2 found from the above calculation can represent the original information system. Using a1a2 as an example, (deleted attributes a3 and a4), we represent our system in Table 1. To obtain the decision rules, one must delete some unnecessary attributes (denoted by *). Table 1 shows four decision rules described as follows: 1) if a1 = 1, then D = 1; 2) if a1 = 3, then D = 2; 3) if a1 = 2 and a2 = 1, then D = 1; 4) if a1 = 2 and a2 = 3, then D = 2.

For the second stage of this study, the KPD affected status and the 12 phenotypes were treated as decision variables. SNP genotypes 11, 12, and 22 were coded as 1, 2, and 3 respectively. The significant conditional attributes retained in the decision rules could be seen as the genes susceptible to the corresponding trait.

### Two-stage method

At the first stage, based on the phenotypes of subjects and their parents, classification trees were built to predict trait values. Phenotypes retained in the decision trees were then advanced to the second stage where RST was applied to discover the minimal subsets of genes associated with the disease trait. For comparison, decision trees were also constructed to map susceptible genes at the second stage. In addition, phenotypes not significantly associated with the KPD affected status were also analyzed at the second stage. Analysis was done for each of the four groups as well as for the pooled data of the four groups. SNPs on a same chromosome were analyzed at the same time. Genome scans were performed by analyzing the pooled set of the significant SNPs across the 10 chromosomes.

## Results

### Relationships between KPD and 12 phenotypes

**Figure 1:**
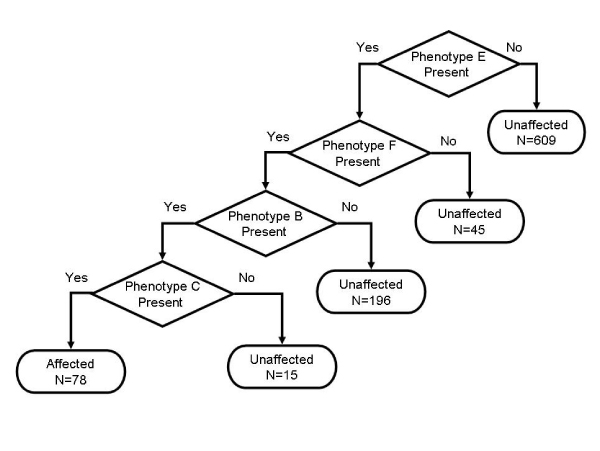
The decision trees by phenotypes for predicting the disease trait based on data from New York City in the first replicate.

At the first stage, the decision trees had accuracy rates of about 99% in predicting the disease trait. The set of significant phenotypes for predicting the disease trait varied among groups. This might suggest some population-specific effects with respect to KPD. The rest of the first ten replicates showed similar results. Phenotypes *b *and *h *were the most common effects shown in the decision trees across groups (6 out of 10, and 7 out of 10, respectively). More nodes were needed to construct decision trees in the NYC group than in the other three groups (Figure 1). In addition, the decision trees remained the same when the parental phenotypes, one at a time or 12 at the same time, were taken into consideration in the construction of the trees.

### SNPs identified to be associated with KPD and phenotypes

**Figure 2:**
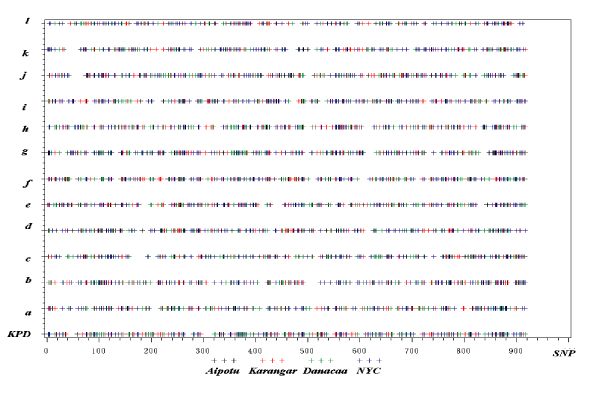
SNPs related to traits in Aipotu, Karangar, Dancaa, and NYC by rough set theory.

Figure 2 showed the SNPs identified to be associated with KPD and the 12 phenotypes in each of four groups using RST. Genotypes that were significantly associated with KPD did not show better results in terms of hits of true susceptible SNPs. Similar result were found in the rest of the first 10 replicates.

## Discussion

In this study, the decision trees based on a few phenotypes successfully predicted the KPD affected status at the first stage. Some phenotypes were frequently included in the decision trees. These phenotypes might be used to screen KPD or to become biomarkers themselves. The decision trees for the NYC group had different structures, in terms of number of nodes. This might be due to the underlying genetic background or the extended pedigree structures. Further study should clarify the difference. It was difficult to rationalize the failures of the application of decision trees and RST methods in identifying SNPs susceptible to KPD or the 12 phenotypes. One possible reason was that the two methods might not be suitable to decompose the complex algorithms of the simulation. Another conjecture was the low penetrance rate of the disease alleles. It is also possible that association does not exist in this kind of population with SNPs so far apart. It would be interested to estimate statistical power of the two methods (RST and decision trees) for identifying SNPs with various levels of penetrance rates.

## Conclusion

Our results showed that the decision trees at the first stage had accuracy rates about 99% in predicting the disease trait. The application of the decision trees and RST failed to identify some disease-related loci.

## Abbreviations

GAW14: Genetic Analysis Workshop 14

KPD: Kofendrerd Personality Disorder

RST: Rough set theory

## Authors' contributions

L-YW participated in the study design, carried out the analysis, and drafted the manuscript. C-LH participated in the study design, developed the algorithms, and helped to draft the manuscript. C-HC participated in study design and coordination and helped to draft the manuscript. All authors read and approved the final manuscript.

## Figures and Tables

**Table 1:** An example of application of the rough set theory

Row data						Decision rules			
	
Subject	a1	a2	a3	a4	D	Subject	a1	a2	D
X_1_	1	2	1	1	1	x1	1	2	1
X_2_	1	3	1	3	2	x2	*^a^	3	2
X_3_	2	3	1	2	2	x3	*	3	2
X_4_	3	1	1	3	1	x4	*	1	1
X_5_	3	2	1	3	2	x5	3	2	2
X_6_	3	1	1	1	1	x6	*	1	1
X_7_	1	1	2	2	1	x7	*	1	1
X_8_	2	3	2	1	2	x8	*	3	2

^a^*, unnecessary attribute.

**Table 2:** A discernibility matrix of the rough set theory

Subject	X_1_	X_2_	X_3_	X_4_	X_5_	X_6_	X_7_	X_8_
X_1_	-^a^							
X_2_	a2, a4	-						
X_3_	a1,a2,a4	-	-					
X_4_	-	a1,a2	a1,a2,a4	-				
X_5_	a1, a4	-	-	a2	-			
X_6_	-	a1,a2,a4	a1,a2,a4	-	a2,a4	-		
X_7_	-	a2,a3,a4	a1,a2,a3	-	a1,a2,a3,a4	-	-	
X_8_	a1,a2,a3	-	-	a1,a2,a3,a4	-	a1,a2,a3	a1,a2,a4	-

^a^-, the two elements belonged to the same class so the set of attributes that discerns the two elements were omitted.
